# The Neuroprotective Effect of Carvedilol on Diabetic Neuropathy: An *In Vitro* Study

**DOI:** 10.1155/2021/6927025

**Published:** 2021-01-16

**Authors:** Rania M. Magadmi, Mujahid A. Alsulaimani, Aziza Rashed Al-Rafiah, Ahmed Esmat

**Affiliations:** ^1^Department of Pharmacology, Faculty of Medicine, King Abdulaziz University, Jeddah, Saudi Arabia; ^2^Neuroscience Unit, Faculty of Medicine, King Abdulaziz University, Jeddah, Saudi Arabia; ^3^Department of Pharmacy, Ministry of Health, Taif, Saudi Arabia; ^4^Medical Laboratory Technology Department, Faculty of Applied Medical Sciences, King Abdulaziz University, Saudi Arabia; ^5^Department of Pharmacology and Toxicology, Faculty of Pharmacy, Ain Shams University, Cairo, Egypt

## Abstract

Diabetic neuropathy serves as a major complication for diabetic patients across the world. The use of effective treatment is integral for reducing the health complications for diabetic patients. This study has evaluated the carvedilol potential neuroprotective effect on diabetic neuropathy. An *in vitro* model of diabetic neuropathy was used, including dorsal root ganglia (DRG) that were cultured from male adult mice C57BL. These were incubated for about twenty-four hours in high glucose (HG) media (45 mM). Some cells were incubated with carvedilol (10 *μ*M). Neuronal viability, neuronal morphology, and activating transcription factor 3 (AFT3) were measured. The cell viability was decreased, along with neuronal length, soma area, and soma perimeter with HG media. Also, there was an overexpression of ATF3, which is a neuronal stress response marker. The pretreatment with carvedilol increased the viability of DRG as compared to HG-treated cells. Also, it significantly protected the DRG from HG-induced morphology changes. Though it shows a decrease in AFT3 expression, the statistical results were insignificant. The current study demonstrates the neuroprotective effect of carvedilol against HG-induced DN using an *in vitro* model. This could be through carvedilol antioxidant effects.

## 1. Introduction

The prevalence of diabetic neuropathy (DN) is recognized to affect about 50 percent of diabetic patients (both type 1 and type 2). Most studies confirm the DN as the major reason for diabetic patient's hospitalization as well as nontraumatic amputations [1]. Callaghan et al. [2] state that the management of DN is generally attained through appropriate blood glucose levels within the normal range and symptomatic therapy. Although various researches have explored DN chronic pain management, only a few improvements are achieved, where the remaining fail to meet the clinical needs of DN [3]. Tesfaye [4] noted that only two compounds show a clinical improvement, namely, *α*-lipoic acid and epalrestat. Tesfaye [4] and Kumar and Mitta [3] stated that effective medications need to be developed to prevent DN progression.

Carvedilol is an *α* and *β* adrenoreceptors blocker and has peripheral vasodilators effect by blocking the *α*1 adrenoreceptor [5, 6]. It is often prescribed for the treatment of chronic heart failure, hypertension, and left ventricle dysfunction after myocardial infarction. It also helps to block the oxygen radicals to damage nitric oxide-mediated coronary artery vasodilation [7]. Liu and Wang [8] and Diogo et al. [9] further emphasized the neuroprotective effects of carvedilol due to its antioxidant property. Areti et al. [10] further show the tendency of carvedilol to counteract the oxaliplatin-induced oxidative stress in neuronal cells.

These properties of the carvedilol make it suitable for use in the therapeutic treatment of DN patients. Thereby, this study is aimed at investigating the potential protective effect of carvedilol using *in vitro* model of DN.

## 2. Material and Methods

### 2.1. Ethical Approval

This study followed all ethical guidelines related to the handling of animals and *in vitro* procedures. Also, the ethical committee at the Faculty of Medicine, at King Abdulaziz University (Reference No. 237-18), approved this study. The researcher ensured the practice of procedures that minimized the suffering of the animals.

### 2.2. DRG Isolation and Culture

Primary adult dorsal root ganglion (DRG) cultures were used as *in vitro* model of sensory neurons. DRGs were isolated from male adult C57BL mice. Cervical dislocation led to the killing of the mice, where the spinal column was cut into halve, at the midline. The spinal column was isolated and put on the phosphate buffer saline (PBS) in the petri dish on ice. The DRGs were collected on PBS. After removing the attached fibers from DRGs, DRGs were incubated with digestive enzymes (0.06 *μ*g/ml of collagenase XI (Sigma) and 0.1 *μ*g/ml of dispase (Sigma) in PBS) at 37°C for one hour, 5% CO_2_. Following it, pipetting was held for triturating the DRG. For neuron selective isolation, a gradient centrifuge technique with 15% bovine serum albumin (BSA) in the medium was used. These were then suspended in neurobasal A media (NBA; Gibco) (with 25 mM glucose) supplemented with 2 mM Glutamax (Gibco), 1% Penicillin/Streptomycin (Gibco), and 2% B-27 supplement (Gibco).

The poly-D-lysine (Sigma, 0.1 mg/ml) was used for coating the well at the mid of the 12-coverslip well plate (or 96-well plates). After this, the plate was incubated at 37°C, 5% CO_2_ for two hours. Following it, poly-D-lysine was removed, and the wells were washed with distal water (three times). Then, each well was added with laminin (Life Technologies, 10 *μ*g/ml) and further incubated for two hours. This laminin was then removed, following which the cells were placed at the center of each well and then incubated overnight.

### 2.3. Induction of *In Vitro* Models of Diabetes Neuropathy

**Figure 1:**
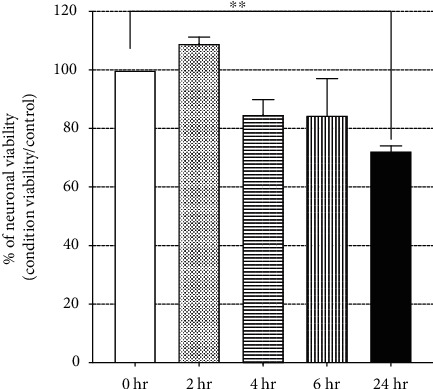
Effect of high glucose medium (45 mM) on DRG culture viability at different time points.

The high glucose (HG) medium (45 mM glucose) was used for the incubation of some DRG cells when the one day has passed after plating had been held for twenty-four hours [11, 12]. The duration of the experiment was decided based on the preliminary data results. The DRG cultures were exposed to HG concentration at different time points (2, 4, 6, and 24 hours), following which the cell viability is assessed (Figure 1). The DRG viability had substantially decreased after a day of being exposed to HG exposure to 72% (*p* = 0.0086). Thus, each group culture was incubated for 24 hours before the experiment.

**Figure 2:**
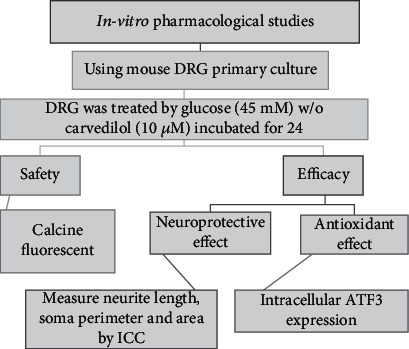
Procedure followed in this *in vitro* study.

The DRG culture was divided into 4 groups. The control group had standard medium glucose concentration for optimum neuron growth (25 mM), HG group (45 mM), and HG media (45 mM) plus carvedilol (Roche, 10 *μ*M) [10, 13]. Finally, the reference group has (45 mM) medium plus *α*-lipoic acid (100 *μ*M) [14]. The carvedilol and lipoic acid were dissolved in dimethyl sulfoxide (DMSO). Then, the safety and efficacy of carvedilol were assessed as shown in Figure 2.

### 2.4. Viability Assay

**Figure 3:**
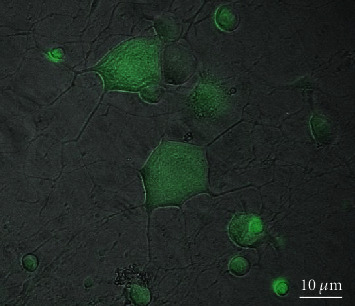
Calcein-AM-stained fluorescent image of the DRG culture.

Calcein-AM fluorescent assay kit was used for cell viability assessment (Vibrant cell assay kit, CAT#V13181, Invitrogen, Life Technologies, USA). It is a nonfluorescent lipophilic dye. This is cleaved using the endogenous esterases to calcein, intracellularly, a highly green fluorescent (Figure 3). It is considered a viability maker because it is quenched upon the loss of cell viability. DRG cells were cultured in a 96-well plate with the corresponding drugs for 24 hours before performing the viability test. As per company protocol, 5 *μ*m of calcine was added to each well and incubated for 45 minutes at 37°C in the dark. Thereafter, cell viability was measured by a fluorescent microplate reader (BioTek®, Synergy HT, USA) at excitation and emotion of 480/520, respectively. Experiments were run in triplication. Background fluorescence from DRG-only wells was subtracted. The viability was presented as the percentage of living cells to the control group.

### 2.5. Immunocytochemistry

DRG immunocytochemistry was performed using a 12-well plate. Phosphate buffer saline (PBS) was used to wash the cells thrice, after which these were incubated with 4% paraformaldehyde for ten minutes. Following it, paraformaldehyde was removed and PBS was used to wash the cells; Triton X-100 (Sigma Aldrich (MERCK), USA) (0.25%) was used for enhancing the dye permeability across the cell membrane and left for ten minutes at room temperature. Then, the cells were incubated with blocking buffer (2% goat serum (Life Technologies, USA), 0.2% fish serum gelatine (Sigma, G7765), and 0.025% Triton X-100 in PBS for one hour at room temperature. These were then stained with primary antibody (mouse anti-B III tubulin monoclonal IgG; R&D MAB1195 clone TuJ-1; 1 : 1000 in blocking buffer), overnight at 4°C. Followed by the removal of the primary antibody, a blocking buffer was used to wash the cells thrice. The incubation of the cells was held for secondary antibody Alexa Fluor® 594 goat antimouse (Invitrogen A-21203, 1 : 1000) for two hours in the dark at room temperature. Following it, the removal of secondary antibodies was held with thrice washing with PBS. The cells' nuclei were stained by 4′, 6-diamidino-2-phenylindole (DAPI) (Invitrogen, P36935, USA). Each condition was presented with two coverslips, where the experiments were repeated thrice. 20x and 40x objectives of the fluorescent microscope (Nikon Y-TV55, Japan) were used for capturing images. The Fiji-ImageJ [15] software was used for image analysis.

### 2.6. DRG Morphology Analysis

Antitubulin III antibody was used for the staining of the DRG cells for detecting the neurons and neuritis. The ImageJ software was used for different random sections to measure the neurite length, soma area, and perimeter. Neurite length was measured by tracing each neurite to identify the total number of neurite length (in *μ*m), then divided over the number of neurons present in the section. The soma area and perimeter were measured by using the freehand selection tool around the edge of each neuron.

### 2.7. Evaluation of Neuronal Stress Response in DRG Culture

The expression of activation transcription factor 3 (ATF3) was used for evaluating HG-induced stress in cultured DRG [16]. Conjugated ATF3 antibody (ATF3, Polyclonal, bs-0519R, Bioss Antibodies, USA; 1 : 100) was used for cells staining. Fluorescent microscopy was used for imaging of cells at power of 20X. These experiments were conducted in triplication. Negative control was used for non-treated cells. ATF3 percentages were calculated for positive neurons and compared between the groups.

### 2.8. Statistical Analysis

The findings were presented in the form of mean ± standard error (SE). One-way Analysis of Variance (ANOVA) was used to make comparisons to the HG group. Each experiment was repeated three times in duplication. The GraphPad Prism version 8 software (GraphPad Software, La Jolla, CA, United States) was used for statistical analysis. The statistical significance was set at 0.05.

## 3. Results

### 3.1. Effect of Carvedilol on HG-Induced Decreases in DRG Viability

**Figure 4:**
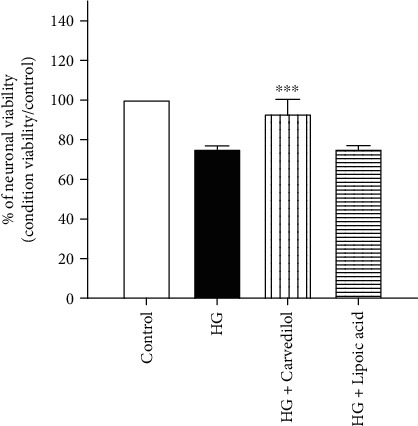
Carvedilol effect on HG-induced decreases in DRG viability.

In order to evaluate the neuroprotective effect of carvedilol in diabetic neuropathy, DRG cultures with HG media were incubated with or without carvedilol.  Figure 4 shows the percentage of cell viability between different groups. The percentage of viability was calculated relative to negative control as 100% viability. The DRGs exposed to HG media showed more than 20% significantly decreased cell viability compared to the corresponding control (*p* = 0.0001). The neurons in HG media were found to be 75% viable using a calcein live-dead viability kit. However, 10 *μ*M of carvedilol significantly increased the DRG viability by more than 18% compared to the HG group (*p* = 0.001).

### 3.2. Effect of Carvedilol on Neuronal Morphology in HG-Exposed DRG

To investigate the protective effect of carvedilol on neuronal morphology, DRG cultures were incubated with HG with and without carvedilol. Then, cultures were stained with anti-*β* III tubulin Ab to visualize the neurons and neurites. Finally, the neuron perimeter, area, and neurite length were measured.

### 3.3. Neurite Length

**Figure 5:**
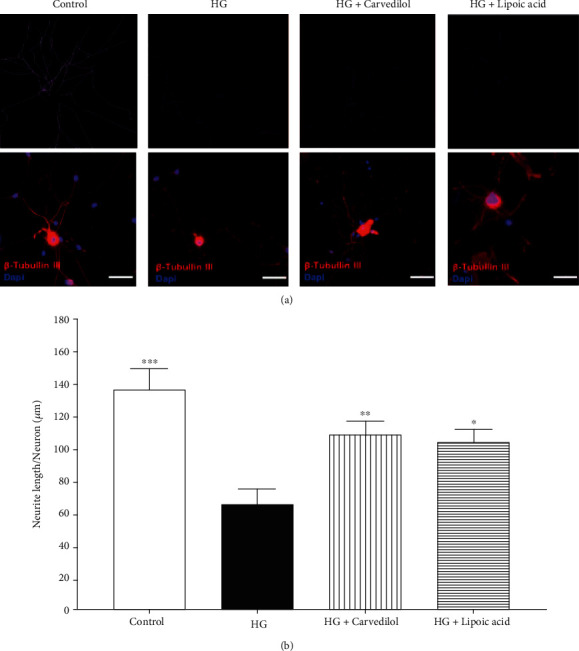
Effect of the carvedilol on neurite length in high-glucose media.

As shown in Figure 5, the DRG exposed to HG for 24 hours showed a significant reduction in neurite length compared to control cells (65.08 *μ*m and 136.9 *μ*m, respectively; *p* < 0.001). However, 10 *μ*M of carvedilol prevented HG-induced reduction in neurite outgrowth by about 60%. Also, the *α*-lipoic acid showed the same improvement effect on the neurite length.

### 3.4. Soma Perimeter

**Figure 6:**
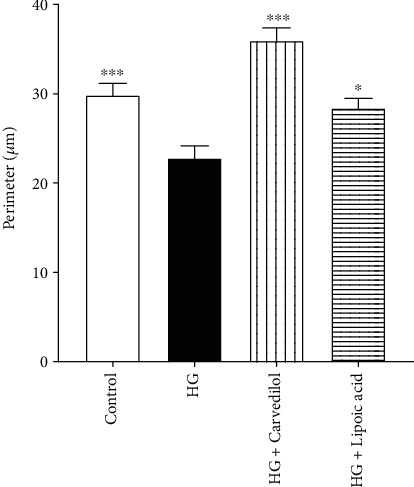
Effect of the carvedilol on soma perimeter in high-glucose media.

As shown in Figure 6, there was a significant decrease (*p* < 0.001) in the soma perimeter of the cells exposed to HG media compared to the corresponding control (from 29.85 *μ*m to 22.78 *μ*m). Carvedilol (10 *μ*M) was able to improve the neuronal perimeter by 58% compared to the HG group. Interestingly, *α*-lipoic acid has the same effect on neuronal perimeter shown in the cells treated with carvedilol.

### 3.5. Soma Area

**Figure 7:**
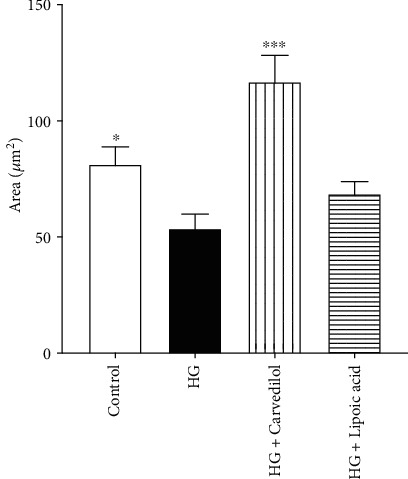
Effect of the carvedilol on soma area in high-glucose media.

As shown in Figure 7, the area of neurons was significantly decreased more than 1.5-fold in response to HG (*p* = 0.02). In contrast, 10 *μ*M of carvedilol could protect the reduction in the area of the neurons by more than 2-fold compared with high glucose (*p* < 0.001) and more than the control. Remarkably, lipoic acid improved the reduction of the soma area but did not reach a statistically significant level (*p* = 0.52).

Taken together, these results demonstrate that carvedilol could protect against morphological changes induced by HG in neuronal cells.

### 3.6. Effect of Carvedilol on ATF3 Expression on Neurons

**Figure 8:**
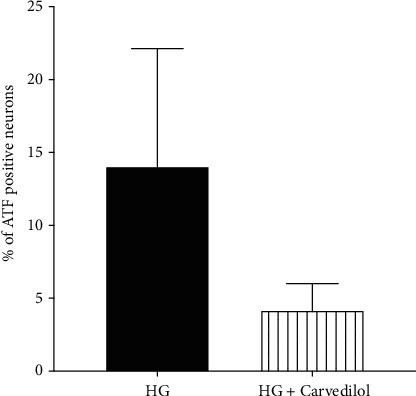
Percentage of ATF-positive neurons in high-glucose DRG cultures.

To investigate the mechanism of the neuroprotective effect of carvedilol, the neuronal stress marker (ATF3) expression was evaluated. 10 *μ*M of carvedilol decreased the ATF3 expression in HG-treated DRGs, but this did not achieve statistical significance (*p* = 0.115) as shown in Figure 8.

## 4. Discussion

Diabetes mellitus affects around 463 million people worldwide and is the most common cause of neuropathy. Several mechanisms could explain the pathogenesis of hyperglycemia to induce microvascular complications in diabetic patients. Oxidative stress could play an important role in cellular injury [17]. Recently, carvedilol has also been appreciated for its antioxidant activity [16, 18–20]. Moreover, diabetes and hypertension are common comorbidities specialty in advanced age patients [21]. The incidence of hypertension increases among diabetic patients [22] and vice versa [23]. Taken together, those attractive properties of carvedilol make it a suitable therapeutic candidate for managing diabetic neuropathy. Therefore, the neuroprotective impact of carvedilol has been examined using the *in vitro* model of DN.

The use of *in vitro* method was based on providing new insights. Such as according to Vincent et al. [14], the study of peripheral neuropathy and DN uses DRG extensively. According to Melli and Höke [24], DRG culturing could be cell lines. Melli and Höke [24] examined that the cell line is inferior as compared to the primary cell culture as it gives fewer stimuli of an *in vivo* biological method. Melli et al. [25] assessed the benefits of adult rodent DRG culturing as they do not require serum and NGF in their growth, unlike embryonic DRG culture.

In the current study, the neurite length and soma area and perimeter also decreased with the induction of HG media. Similar results in previous studies showed that the short-term exposure to HG media caused a reduction in the neurite outgrowth in primary DRG cultures [26]. They contributed this effect as the increase of reactive oxygen species (ROS) generation and the decrease in the GSH level [26]. An increase in ROS generations after exposed to HG concentration is time-dependent [26]. An increase in ROS and intracellular Ca regulate and release the proapoptotic factors leading to altering the neuron morphology and subsequently cells death [26]. Moreover, HG induces swelling in mitochondria depolarization in sensory neurons leading to depletion in ATP and subsequently apoptosis. Thus, the oxidative stress represents a target for the neuroprotective effect of therapeutic intervention in diabetic neuropathy. Therefore, carvedilol was an attractive candidate to prevent diabetic neuropathies.

In the current study, carvedilol significantly improved neuronal survival and neurite length in HG media. Consistent with these results, a previous study showed that carvedilol concentration from 0.1 to 20 *μ*M significantly protects cortical neuronal cultures from cytotoxicity during hypoxia and reoxygenation model [27]. Moreover, 10 *μ*M of carvedilol prevents oxaliplatin-induced neurotoxicity in a neuronal cell line (N2a) by significantly reduced ROS production [10]. However, no previous *in vitro* work has been done to evaluate the carvedilol effect in the diabetic neuropathy model. To the best of our knowledge, this is the first *in vitro* experiment to demonstrate the neuroprotective effect of carvedilol in diabetes neuropathy.

Remarkably, carvedilol in this study increased the soma diameter and perimeter in HG media even more than the control group. This could be explained by understanding the heterogenicity nature of DRG cultures. DRG contains 3 subtypes of sensory neurons: A*β*-fibers, A*δ*-fibers, and C-fibers. These fibers are classified based on their conduction velocity which positively correlates with the diameter and degree of myelination. The largest diameter fibers with thick myelination are A*β*-fibers. They have the fastest conduction velocity, and they are classified as nonnociceptive neurons as they respond only to innocuous stimuli such as light touch. A*δ*- and c-fibers are nociceptive neurons with medium and small diameters, respectively [28, 29]. Based on the results from the current study, one can conclude that carvedilol protects the nonnociceptive fibers more than nociceptive fibers. Subsequently, the average of soma diameter and perimeter increased compared to the normal heterogenous population of the control group. However, more immunostaining experiments to visualize the subtypes of preserved neurons in DRG must be carried out to confirm this speculation. Also, the clinical correlation must be considered.

Although the effect of carvedilol on HG-induced oxidative stress in DRG was not directly investigated in this study, the expression of ATF3 was used as a marker of neuronal stress [16, 30, 31]. Wright et al. [31] reported the increase in the expression of ATF3 in sensory neurons in both an *in vitro* and *in vivo* diabetic model. The current study showed that 10 *μ*M of carvedilol reduced, although not reached the statistically significant level, the expression of AFT3 in DRG exposed to HG. This nonsignificant finding could be explained by the relatively small number of neuronal cells in primary DRG cultures. More repeated experiments may be needed to draw a clear conclusion.

Carvedilol exerts antioxidant activity in two distinct ways. These mechanisms can be divided into chemical and biological aspects. Carvedilol could bind chemically and scavenge ROS molecules. Biologically, carvedilol could inhibit the enzymes that generate the ROS, leading to a marked decrease in the ROS production [20]. Evidence from the study supplements the therapeutic efficacy of carvedilol due to its antioxidant and anti-inflammatory activities. Liu and Wang [8] also note the neuroprotective properties of carvedilol, such as it shows improved results for the carvedilol protection of neurons against death.

On the contrary, carvedilol can cover the tachycardia, just like other adrenoreceptor blockers that serve as a warning indication for insulin-induced hypoglycemia in diabetic patients. Likewise, the risk of hypoglycemia and hypoglycemia unawareness can be elevated with the use of beta-blockers by patients with diabetes. However, there is minimal evidence for supporting the establishment that beta-blockers can be routinely contraindicated in diabetes since they have few clinically essential effects on hypoglycemia recovery and unawareness. Symptoms such as sweating might be improved even though some hypoglycemia signs including palpitation and tremor might be blunted [32–35]. The reason is that sweating is a sympathetic cholinergic reaction to hypoglycemia, which cannot be repressed by beta-blockers, and cautious use of beta-blockers improves sympathoadrenal activation by hypoglycemia. Additionally, the total signs of hypoglycemia might not be affected significantly by beta-blockers [36]. Moreover, some studies have recommended that beta-blockers have a minute effect on the risk of hypoglycemia and its risk, specifically from b1-selective beta-blockers. Thereby, diabetic patients should preferentially use selective beta-blockers with cardiovascular diseases not merely to enhance the long-term cardiovascular outcome, but also for potentially life-saving effects throughout severe hypoglycemia [37, 38].

## 5. Conclusion

This study presents the analysis of the neuroprotective effect of carvedilol against *in vitro* HG-induced neuronal damage. Although this study presents effective protection and antioxidant mechanism of carvedilol, more studies are needed so that the molecular mechanism of the antioxidant effect of carvedilol on sensory neurons as well as intracellular signaling can be established. Also, there is a need to establish a human clinical trial for assessing the effectiveness of carvedilol on DN. Since the study was carried out in Saudi Arabia, thereby, Saudi vision 2030 can derive valuable findings from this study, where a potential cure for DN can be discovered for supporting the national economy. Also, the findings could help reduce the mortality, as well as morbidity related to DN along with its related health care expenses. Additional studies are required for evaluating the advantages and disadvantages of beta-blockers for patients with diabetes.

## Data Availability

The datasets used and analyzed during the current study are available from the corresponding author on reasonable request.
